# Laparoscopic Versus Open Colorectal Resection Within Fast Track Programs: An Update Meta-Analysis Based on Randomized Controlled Trials

**DOI:** 10.14740/jocmr2177w

**Published:** 2015-06-09

**Authors:** Qiu-Cheng Lei, Xin-Ying Wang, Hua-Zhen Zheng, Xian-Feng Xia, Jing-Cheng Bi, Xue-Jin Gao, Ning Li

**Affiliations:** a Department of General Surgery, Jinling Hospital, Southern Medical University, Nanjing, Jiangsu Province, China; b Research Institute of General Surgery, Jinling Hospital, Medical School of Nanjing University, Nanjing, Jiangsu Province, China; c Key Laboratory for Medical Molecular Diagnostics of Guangdong Province, Guangdong Medical College, Dongguan, Guangdong Province, China; d Department of Surgery, Prince of Wales Hospital, Faculty of Medicine, the Chinese University of Hong Kong, China

**Keywords:** Fast track programs, Enhanced recovery after surgery, Laparoscopic surgery, Colorectal surgery, Meta-analysis

## Abstract

The objective of the study was to assess the safety and efficacy of laparoscopic colorectal surgery by comparing open operation within fast track (FT) programs. The Cochrane Library, PubMed, Embase and Chinese Biological Medicine Database were searched to identify all available randomized controlled trials (RCTs) comparing laparoscopic with open colorectal resection within FT programs. A total of seven RCTs were finally included, enrolling 714 patients with colorectal cancer: 373 patients underwent laparoscopic surgery and FT programs (laparoscopic/FT group) and 341 patients received open operation and FT programs (open/FT group). Postoperative hospital stay (weighted mean difference (WMD): 0.66; 95% CI: 0.27 - 1.04; P < 0.05), total hospital stay (WMD: 1.46; 95% CI: 0.40 - 2.51; P < 0.05) and overall complications (RR: 1.31; 95% CI: 1.12 - 1.54; P < 0.05) were significantly lower in laparoscopic/FT group than in open/FT group. However, no statistically significant differences on mortality (risk ratio (RR): 2.26; 95% CI: 0.62 - 8.22; P = 0.21), overall surgical complications (RR: 1.19; 95% CI: 0.94 - 1.51; P = 0.15) and readmission rates (RR: 1.33; 95% CI: 0.79 - 2.22; P = 0.28) were found between both groups. The laparoscopic colorectal surgery combined with FT programs shows high-level evidence on shortening postoperative and total hospital stay, reducing overall complications without compromising patients’ safety.

## Introduction

Clear evidence shows that fast track (FT) programs or enhanced recovery after surgery program is beneficial for improving clinical outcomes when compared with traditional care strategies; it can reduce the postoperative morbidity and shorten the hospital stay [1, 2]. This program involves multimodal approaches, including preoperative patient education, optimizing anesthesia, early postoperative enteral nutrition and early mobilization. All these protocols aim to accelerate recovery by attenuating the surgical stress response without compromising patient safety [3, 4].

Over the last decade, FT programs have been widely employed in the perioperative management of colorectal malignancy surgery because of its synergistic positive effect on postoperative outcomes [5]. Anecdotal evidence suggests that FT program may reduce hospital stay and postoperative complications [6, 7]. Meanwhile, minimally invasive surgery is another approach to have such similar function for colorectal patients. Laparoscopic surgery has been generally applied in the treatment of gastrointestinal cancer, which can significantly attenuate tissue trauma, reduce postoperative pain and accelerate the rehabilitation of patients after surgery [8, 9]. Recently, some researchers believe that laparoscopy should also be considered as a key element in the whole FT programs. Combining FT programs and laparoscopic surgery may lead to a faster body physiological recovery and beneficial clinical outcomes; however, no clear conclusion on this has yet been drawn [10-12].

In an effort to clarify the role of combining FT programs and laparoscopic technique in colorectal surgery, Vlug et al [13] conducted a systematic review of two randomized controlled trials (RCTs) and three case-control trials. They showed that no additional benefits of FT pathways were found in laparoscopic colorectal surgery. Oppositely, a meta-analysis including three RCTs with a total of 313 patients indicated a reduction of total hospital stay and postoperative hospital stay for patients in FT programs after laparoscopic surgery. The inconsistent result may be partly associated with the small trials included and thus more randomized studies are needed to clarify the veritable conclusion [14].

The purpose of this present meta-analysis of RCTs is to examine the latest substantive evidence of safety and efficacy for laparoscopic colorectal surgery when compared with the open operation within FT programs.

## Methods

### Literature search

The Cochrane Library, PubMed, Embase and Chinese Biological Medicine Database were searched to identify all RCTs of interest from January 1990 to December 2014. The following terms were used: “fast-track”, “enhanced recovery”, “multimodal perioperative care”, “multimodal rehabilitation”, “laparoscopy”, “laparoscopic”, “colorectal”, “colon”, “rectal”, “open surgery”, and “laparotomy”. Review articles were used to identify additional relevant studies. Only eligible full texts in English or Chinese were considered for review.

### Inclusion and exclusion criteria

The inclusion criteria were as follows: 1) patients with malignant or benign colorectal diseases; 2) comparing laparoscopic with open colorectal resection within the setting of an FT program; 3) studies reporting any of the following outcomes: mortality, postoperative hospital stay, total hospital stay, overall complications, surgical complications and readmission rates; 4) when some studies were reported by the same authors, they were selected only if there was no overlap between the results of their researches; 5) randomized controlled trials. The exclusion criteria were as follows: 1) case reports and review; 2) non-comparative articles; 3) studies reporting the same patient cohorts evaluated in the published literature.

### Study selection and data extraction

Two authors independently selected relevant studies based on title and abstract and extracted data using a double-extraction method for eligibility according to our inclusion criteria. Primary outcomes were mortality and postoperative hospital stay. Secondary outcomes were total hospital stay, overall complications, postoperative complications and readmission rates. A pre-specified subgroup analysis of surgical complications was performed to analyze the potential sources of heterogeneity. Total hospital stay was defined as postoperative hospital stay plus readmission stay. Two reviewers independently performed the literature search, evaluation of trials, quality assessment and data extraction. Discrepancies were resolved by discussion and by consensus with a third party.

### Statistical analysis

Analysis was performed by using Revman 5.2 software (Cochrane IMS, Oxford, UK). If the included study provided medians and inter-quartile ranges instead of means and SD, we imputed the means and SD as described by Hozo et al [15]. And we also calculated the lower and upper ends of the range by multiplying the difference between the median and upper and lower ends of the inter-quartile range by 2 and adding or subtracting the product from the median [14, 16]. The outcomes for categorical variables were aggregated to obtain a pooled risk ratio (RR) with the 95% confidence interval (CI). The pooled effect for continuous variables was applied as weighted mean difference (WMD) with the corresponding 95% CI. Heterogeneity of the mean difference was assessed using I^2^ and x^2^ tests with a P < 0.1 and I^2^ > 50% considered to be significant. A random effects model was adopted for meta-analysis of the outcomes and a P value less than 0.05 was considered to be significant.

### Methodological quality

The quality of all included RCTs was assessed by two researchers using the Cochrane risk of bias tool [17], which includes seven specific items such as random sequence generation, allocation concealment, blinding of participants, personnel and outcome assessors, incomplete outcome data, selective outcome reporting and others.

## Results

### Search results

**Figure 1:**
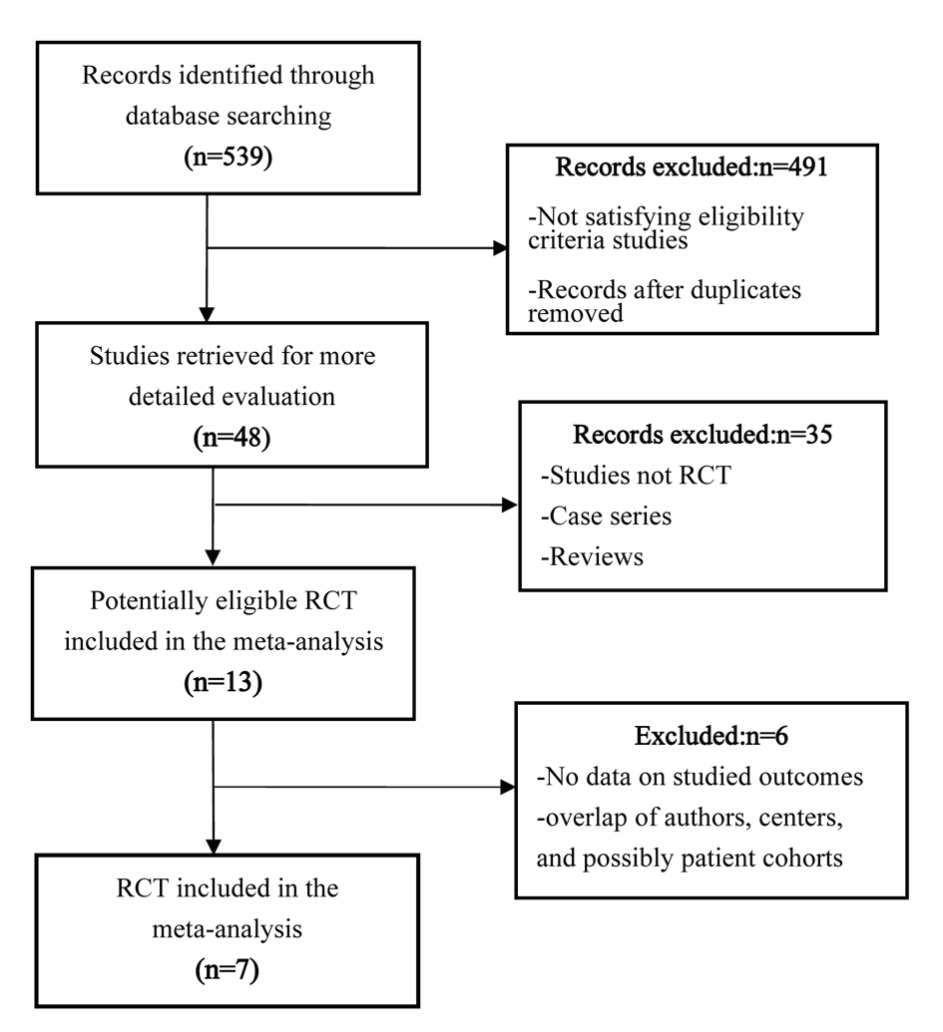
Flow chart illustrating the study selection process.

A total of 539 records were identified through the database search, and 491 were excluded because of the irrelevant objectives and duplicates of the available literatures. Of the 48 potentially relevant records screened, 13 met the selection criteria for the current meta-analysis. Two out of 13 studies did not report available data of the related outcomes, and four studies were excluded because of the overlap of authors, centers, and possibly patient cohorts. Finally, seven RCTs were included for the meta-analysis (Fig. 1).

### Study characteristics and methodological quality

**Figure 2:**
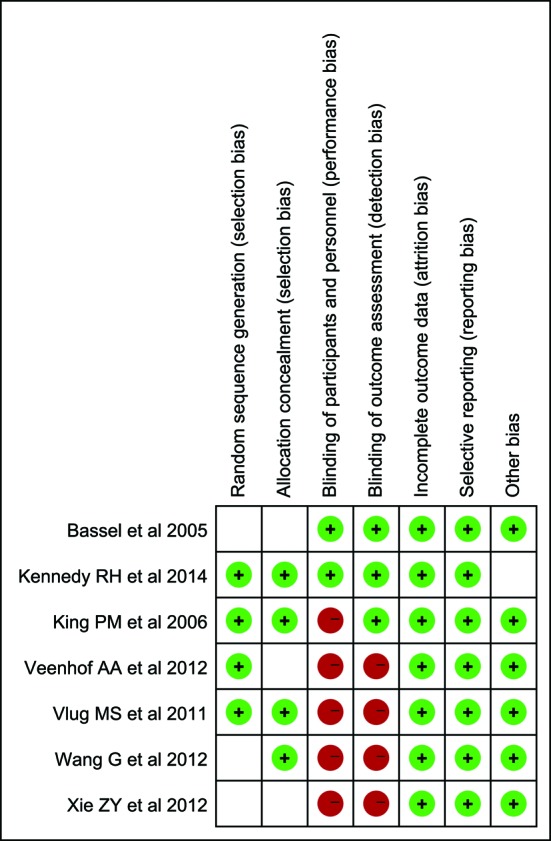
Risk of bias summary: review authors’ judgments about each risk of bias item for each included study.

Characteristics of the seven RCTs [10, 11, 18-22] included in the current meta-analysis were presented in Table 1 and Table 2. All studies were published between 2005 and 2014 and investigated a total of 714 patients: 373 underwent laparoscopic and FT programs; 341 received open operation and FT programs. Figure 2 summarized the risks of bias on the included studies, most of which were moderate qualities.

**Table 1:** Study Characteristics for the Included Studies

Trail	Year	Cancer	No. of patients	Patients (O/L)	Mean age (O/L) (years)	Resection type	Follow-up
Basse et al [10]	2005	Colon	60	30/30	75/75.5	RH; SR	30 days
King et al [11]	2006	Colon and rectum	60	19/41	70.4/72.3	LH; RH; SR; AR; APR	42 days
Vlug et al [18]	2011	Colon	193	93/100	66/66	LH; RH	30 days
Wang et al [19]	2012	Colon	81	41/40	57.2/55.7	LH; RH; SR	30 days
Veenhof et al [20]	2012	Colon	36	17/19	65/65	LH; RH	NR
Xie et al [21]	2012	Colon and rectum	80	40/40	61.3/60.4	LH; RH; APR	30 days
Kennedy et al [22]	2014	Colon and rectum	204	101/103	70.1/69.3	LH; RH; AR; APR; other rectum and colon surgery	> 1 year

O/L: open surgery group/laparoscopic surgery group; NR: not reported; RH: right hemicolectomy; SR: sigmoid resection; LH: left hemicolectomy; RH: right hemicolectomy; AR: anterior resection; APR: abdominoperineal resection.

**Table 2:** Key Features of the FT Programs for the Included Studies

Study	Avoid bowel prep	Standard laxatives	Carbohydrate loaded	Fluid restriction	Standard anesthetic	Early feeding	Early mobilization	Other	No. of ERAS elements
Basse et al [10]	+	+	-	+	+	+	+	+	7
King et al [11]	+	+	-	+	+	+	+	+	12
Vlug et al [18]	-	-	+	-	+	+	+	+	15
Wang et al [19]	+	-	+	+	+	+	+	+	10
Veenhof et al [20]	-	-	+	-	+	+	+	+	15
Xie et al [21]	+	-	-	+	+	+	+	+	10
Kennedy et al [22]	-	+	+	+	+	+	+	+	18
Total	4	3	4	5	7	7	7	7	Total

### Primary outcomes

#### Mortality

Five out of seven RCTs [10, 11, 18, 19, 22] reported the mortality rates. The mortality in included studies was 9 (3.2%) in open/FT group (open operation and FT programs), while 3 (1.0%) in laparoscopic/FT (laparoscopic surgery and FT programs) group; it decreased to 1.0%. However, no significant difference was found between both groups (RR: 2.26; 95% CI: 0.62 - 8.22; P = 0.21; Table 3). There was no significant heterogeneity between these studies (x^2^ = 0; P = 0.50; I^2^ = 0%).

**Table 3:** Comparisons of Outcomes Between Laparoscopic and Open Colorectal Resection Within FT Programs

Outcome	Studies	Number	Effect estimate (95% CI)	Heterogeneity	Test for overall effect
Mortality	5	598	RR = 2.26 (0.62 - 8.22)	x^2^ = 0, I^2^ = 0%, P = 0.50	P = 0.21
Overall complications	7	714	RR = 1.31 (1.12 - 1.54)	x^2^ = 6.00, I^2^ = 0%, P = 0.42	P = 0.0007
Readmission rates	6	678	RR = 1.33 (0.79 - 2.22)	x^2^ = 6.24, I^2^ = 20%, P = 0.28	P = 0.28

The P values of comparisons are given in the column labeled “Test for overall effect”.

#### Postoperative hospital stay

**Figure 3:**
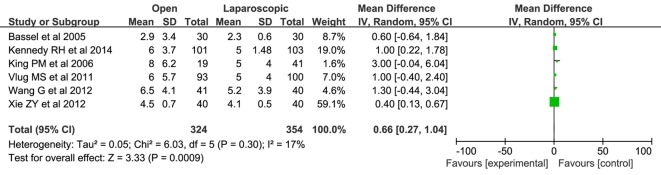
Forest plots of pooled estimates on postoperative hospital stay. CI: confidence interval; df: degrees of freedom; MH: Mantel-Haenszel (statistical method).

A pooled analysis of six articles [10, 11, 18, 19, 21, 22] enrolling 678 patients observed the postoperative hospital stay. A significant reduction in the laparoscopic/FT group was found when compared with open/FT group (WMD: 0.66; 95% CI: 0.27 - 1.04; P = 0.0009; Fig. 3). There was no significant heterogeneity among these studies (x^2^ = 6.03; P = 0.30; I^2^ = 17%).

### Secondary outcomes

#### Total hospital stay

**Figure 4:**
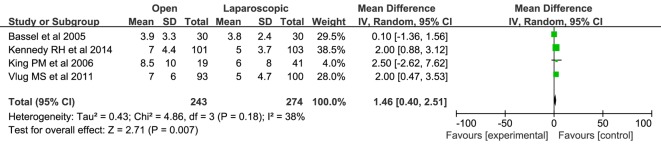
Forest plots of pooled estimates on total hospital stay. CI: confidence interval; df: degrees of freedom; MH: Mantel-Haenszel (statistical method).

Only four RCTs [10, 11, 18, 22] (517 patients) reporting the total hospital stay were pooled. The total hospital stay was significantly shorter in the laparoscopic/FT patients than that observed in open/FT (WMD: 1.46; 95% CI: 0.40 - 2.51; P = 0.007; Fig. 4). There was no significant heterogeneity among these studies (x^2^ = 4.86; P = 0.18; I^2^ = 38%).

#### Overall complications

All seven trials [10, 11, 18-22] (714 patients) reported the rate of overall complications following colorectal surgery. Forty-six point nine percent patients (160/341) in the open/FT group and 34.6% patients (129/373) in the laparoscopic/FT group developed complications respectively. Pooled results detected a statistical difference between both groups (RR: 1.31; 95% CI: 1.12 - 1.54; P = 0.0007; Table 3). There was no significant heterogeneity in these studies (x^2^ = 6.00; P = 0.42; I^2^ = 0%).

#### Surgical complications

**Figure 5:**
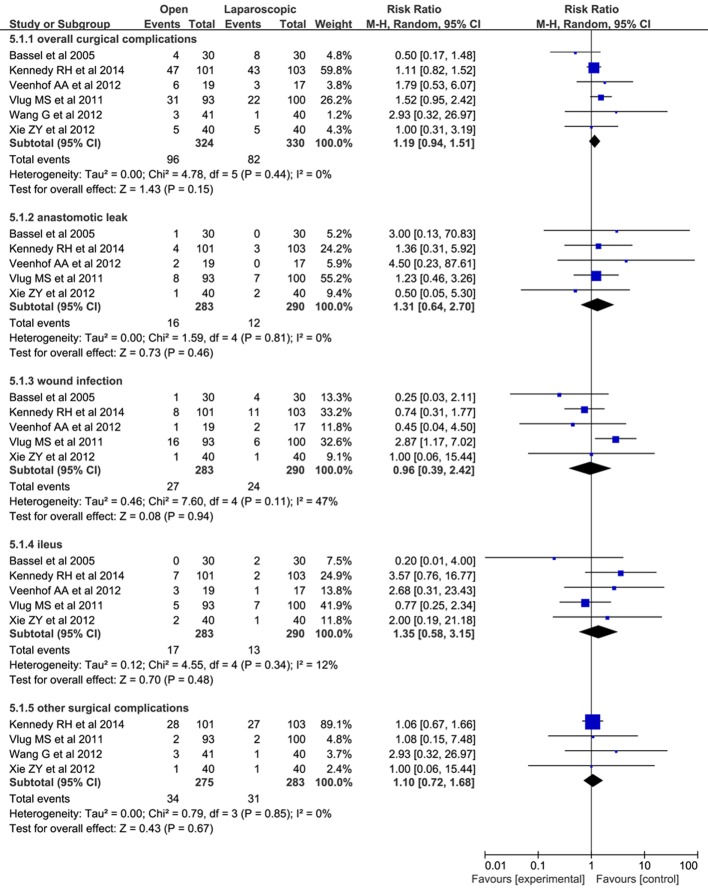
Forest plots of pooled data on surgical complications. CI: confidence interval; df: degrees of freedom; MH: Mantel-Haenszel (statistical method).

Six studies [10, 18-22] with 324 subjects in open/FT groups and 330 subjects in laparoscopic/FT groups investigated the rates of surgical complications. The pooled results indicated that there was no significant difference on surgical complications rates between both groups (RR: 1.19; 95% CI: 0.94 - 1.51; P = 0.15; Fig. 5). No significant heterogeneity was found among these studies (x^2^ = 4.78; P = 0.44; I^2^ = 0%). In the subgroup analysis, there was no significant heterogeneity among subgroup studies (Fig. 5). The results of subgroup analysis are as follows.

#### Anastomotic leak

Data were collected from five studies (573 patients) on anastomotic leak for open/FT vs. laparoscopic/FT [10, 18, 20-22]. Five point seven percent (16/283) had an anastomotic leak in the open/FT group and 4.1% (12/290) in the laparoscopic/FT group. Pooling the results indicated that both groups had similar risks of anastomotic leak (RR: 1.31; 95% CI: 0.64 - 2.70; P = 0.46).

#### Wound infection

Data were collected from five studies (573 patients) on wound infection for open/FT vs. laparoscopic/FT [10, 18, 20-22]. Nine point five percent (27/283) had wound infections in the open/FT group and 8.3% (24/290) in the laparoscopic/FT group. Pooling the results indicated that laparoscopic/FT did not significantly reduce wound infections compared with open/FT (RR: 0.96; 95% CI: 0.39 - 2.42; P = 0.94).

#### Ileus

Data were collected from five studies (573 patients) on anastomotic leak for open/FT vs. laparoscopic/FT [10, 18, 20-22]. Six percent (17/283) had ileus in the open/FT group and 4.5% (13/290) in the laparoscopic/FT group. Pooling the results indicated no significant difference in the risk of ileus (RR: 1.35; 95% CI: 0.58 - 3.15; P = 0.48).

#### Other surgical complications

Data were collected from four studies (558 patients) on other surgical complications for laparoscopic/FT vs. open/FT [18, 19, 21, 22]. Twelve point four percent (34/275) had other surgical complications in the open/FT group and 11.0% (31/283) in the laparoscopic/FT group. Pooling the results indicated no apparent difference in other surgical complications (RR: 1.10; 95% CI: 0.72 - 1.68; P = 0.67).

#### Readmission rate

Six studies [10, 11, 18, 19, 21, 22] (678 patients) reported this outcome. Thirty-five patients from the open/FT group and 30 patients in the laparoscopic/FT group had to be readmitted, and the pooled results detected no statistical difference between groups (RR: 1.33; 95% CI: 0.79 - 2.22; P = 0.28; Table 3). There was no significant heterogeneity among these studies (x^2^ = 6.24; P = 0.28; I^2^ = 20%).

## Discussion

Recently, laparoscopic surgery is considered to be a significant interventional change in traditional care, which leads to improving patients’ clinical outcomes following surgery. Many RCTs have demonstrated a significant reduction of the hospital stay and morbidity for patients with laparoscopic colorectal surgery [23, 24]. The combination of FT programs and laparoscopically assisted colorectal resection has been evaluated in some studies [10-12, 18-20, 25]. However, there were still no clear-out conclusions. A systematic review performed in 2009 showed no robust conclusions between the two compare groups [13]. Most recent meta-analyses only included the RCTs while the controlled clinical trials (CCTs) were excluded. Their results indicated that laparoscopic colorectal surgery was beneficial for reducing the total and postoperative hospital stay in patients receiving FT programs [14].

Our study is the largest meta-analysis that includes more RCTs at present. The results from the present meta-analysis suggested that the implementation of the laparoscopic/FT programs led to a reduction of postoperative and total hospital stay, and the risks of overall complications, surgical complications and readmission were not significantly increased. These results were similar to the meta-analysis by Li et al [14]. This may be accounted for the reason that there were no CCTs included in both two meta-analyses. But the size of samples in our meta-analysis was relatively larger, and it can strongly manifest the beneficial outcomes of laparoscopic/FT programs.

More and more studies have demonstrated colorectal open surgery in combination with FT programs can reduce the length of stay [26, 27]. Similarly, laparoscopic colorectal surgery within traditional care provided comparatively modest benefits compared with open surgery for patients with colorectal cancer [24, 28, 29]. As we know, factors contributing to the prolonged postoperative hospital stay included nausea, pain, and postoperative ileus [7]. In 2005, a Cochrane review has confirmed that laparoscopic colorectal resection brought more safety and decreased the postoperative pain and duration of postoperative ileus than open surgery [30]. The improvement of clinical outcomes in the laparoscopic/FT group may be due to the combining effects of the two approaches [14], which can reduce the postoperative stress response, inflammatory response and enhance rehabilitation [31, 32].

In the terms of safety, our meta-analysis reported a trend toward lower mortality and complication rates for laparoscopic/FT group, and the laparoscopic/FT group has a lower overall complication rate than the open/FT group. Less morbidity was associated with laparoscopic surgery, whereas this was not the case for FT programs [19]. Our study indicated surgical complications were not difference between two groups, but it is noteworthy that different laparoscopic technique levels may affect this outcome. Unlike the previous meta-analysis, we have examined not only the overall complications, but also surgical complications, including anastomotic leak, wound infection, ileus and other surgical complications. All kinds of surgical complications seemed to have higher incidence following open colorectal surgery, but this did not reach statistical significance in comparison with the laparoscopic/FT group.

From the result of our study, it is evident that there is a trend toward lower readmission rates in laparoscopic/FT group, although no significant difference regarding the pooled data could be identified between both groups. The study performed by Li et al [14] showed the similar readmission rate to our review. In addition, it must be realized that this result may be caused by the different discharge criteria of all RCTs.

Another major concern is that laparoscopic technology is more expensive than open surgery. Nevertheless, hospital costs were similar between laparoscopy-assisted procedures and open surgery [33]. However, in a recent non-randomized study [34], total costs were lower with laparoscopic operation, owing to the lower laboratory, pharmacy, and ward nursing costs, although operating room costs were significantly higher after laparoscopic colectomy. In addition, King et al [11] found the cost data were similar between the laparoscopic/FT group and open/FT group. Due to the present lack of data, a cost-effectiveness analysis was not performed in our meta-analysis. However, from the point of view of health economics, we can know that, with the decrease in postoperative and total hospital stay, morbidity and readmission rate, the medical cost for each patient would be significantly lower for those receiving laparoscopic surgery and FT programs than those treated within open operation and FT programs. Take something further, more RCTs need to study the cost problems.

However, some limitations of our study should be noted. First, the overall methodological quality of the included trials was moderate with some sources of bias, especially of the performance and detection bias. Second, a publication bias may exist, which indicated the unpublished studies may influence our results. Third, the sample size of most included studies was small, excepting two studies were large scale multicenter trials, which may have compromised the internal validity.

### Conclusions

In summary, this updated meta-analysis demonstrated that laparoscopic surgery can reduce the postoperative and total hospital stay and overall complications within FT programs without compromising patient’s safety. Further high-quality, multicenter, well-designed RCTs should be conducted to evaluate the effect of laparoscopic surgery within FT programs for colorectal cancer.
